# Structure of Methanol Solvated Iodozinc(II) Complexes in Solution

**DOI:** 10.1007/s10953-018-0737-9

**Published:** 2018-03-19

**Authors:** Ingmar Persson

**Affiliations:** Department of Molecular Sciences, Swedish University of Agricultural Sciences, P.O. Box 7015, 750 07 Uppsala, Sweden

**Keywords:** Zinc(II) iodide, Methanol, Structure in solution, EXAFS

## Abstract

The structures of the solvated zinc ion and the solvated zinc–iodide complexes in methanol solution have been determined by EXAFS. The zinc ion is six-coordinated in an octahedral fashion with a mean Zn–O bond distance of 2.071(4) Å. According to the stability constants of the zinc–iodide system in methanol solution the first complex, ZnI^+^, is suppressed, which may indicate that a coordination change takes place at this step. On the other hand, the second complex, ZnI_2_, predominates at excess of iodide. The methanol solvated ZnI_2_ complex has a tetrahedral structure with mean Zn–I and Zn–O bond distances of 2.55(1) and 1.99(1) Å, respectively. The mean Zn–I bond distance in a solution containing a maximal content of ZnI^+^, ca. 12%, strongly indicates that the first complex also has a tetrahedral structure.

**Electronic supplementary material:**

The online version of this article (10.1007/s10953-018-0737-9) contains supplementary material, which is available to authorized users.

## Introduction

The stability of zinc(II)–iodide complexes varies very much with the solvent used. In some solvents only very weak complex formation is observed, as in water, dimethyl sulfoxide (DMSO) and *N,N*-dimethylformamide (DMF), while in others it can be very strong, as in acetonitrile; the stability constants of the zinc(II)–iodide system in various solvents are summarized in Table 1. A major reason for these large differences in stability of zinc–iodide complexes is the strength of the solvation of the zinc and iodide ions in different solvents. The iodide ion is more strongly hydrated in water than solvated in any of the other solvents where the complex formation of the zinc–iodide system has been studied [10, 11] (Table S1). The zinc ion, on the other hand, is more strongly solvated in solvents such as DMSO, DMF and HMPA (hexamethylphosphoric triamide) than in water, while in solvents such as methanol and especially acetonitrile the opposite is found [12, 13] (Table S1). The strongest complex formation is observed in acetonitrile [9], where the zinc and iodide ions both are weakly solvated [10–12]. The stability of iodozinc complexes in methanol is medium strong as the zinc and iodide ions are both more weakly solvated than in water but more strongly than in acetonitrile.

**Table 1:** Selected reported stability constants of the zinc(II)–iodide system in various solvents with the estimated errors in parenthesis as reported in the original papers

log_10_ *K*_1_	log_10_ *K*_2_	log_10_ *K*_3_	log_10_ *K*_4_	$- \Delta G_{1}^{\text{o}}$	$- \Delta G_{2}^{\text{o}}$	$- \Delta G_{3}^{\text{o}}$	$- \Delta G_{4}^{\text{o}}$	$- \Delta H_{1}^{\text{o}}$	$- \Delta H_{2}^{\text{o}}$	$- \Delta H_{3}^{\text{o}}$	$- \Delta H_{4}^{\text{o}}$	References
*Oxygen donor solvents*												
Water												
− 1.52				− 8.7								[1]
Methanol												
2.81 (2)	4.00(2)			16.0(1)	22.8(1)							[2]
*N*,*N*-dimethylformamide (DMF)												
	4.21(5)^a^	1.65(6)			24.0(3)^a^	9.4(3)						[3]
*N*,*N*-dimethylacetamide (DMA)												
3.8 (1)	3.3(2)			21.7(6)	18.8(1.1)			− 21.3(1.5)	− 2.4(2.0)			[4]
Dimethyl sulfoxide (DMSO)												
− 0.70(10)	1.41(8)	0.15(10)		− 4.0(6)	8.0(7)	−0.9(3)		− 19.0(7.5)	− 29.4(7.8)	− 12.7(2.3)		[5, 6]
Hexamethylphosphoric triamide (HMPA)												
4.06 (17)	1.54(6)			23.2(9)	8.8(4)							[7]
Ethylene glycol												
2.36 (6)	0.68(6)			13.5(3)	3.9(4)							[8]
*Nitrogen donor solvents*												
Acetonitrile												
4.5 (6)	6.69(20)	4.18(7)	2.69(9)	25.7(3.4)	38.2(1.2)	23.9(1.0)	15.4(5)	− 12.0(6.0)	5.2(2.4)	14.7(1.1)	0.0(8)	[9]
Pyridine												
2.45 (4)				14.0(3)				− 20.1(1.1)				[9]

^a^Beta 2

**Fig. 1:**
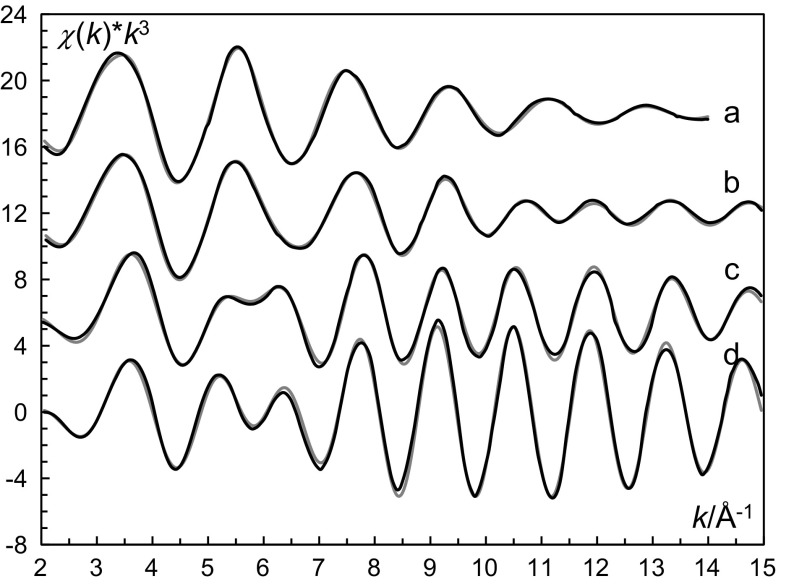
Fit of the *k*^3^-weighted raw EXAFS data for, *a* solution Zn_0, *b* solution Zn_1, *c* solution Zn_2 and *d* solution Zn_4; experimental data—black, line and model with parameters from Table 3—grey line; the solution compositions are given in Table 2

The complex distribution of the zinc–iodide systems in the solvents studied show two principal patterns with the first complex dominant over a wide range of free iodide concentration or almost completely suppressed (Figs. S1 and S2); the complex formation function of the zinc–iodide system in methanol is given in Fig. S3. The common property of the solvents where the monoiodozinc complex disproportionates into the solvated zinc ion and the solvated diiodozinc complex, is that the solvated zinc ion is six-coordinate in an octahedral fashion (Table S2). The solvents where the monoiodozinc complex has a wide range of existence, HMPA and ethylene glycol, are both space-demanding upon coordination. The HMPA solvated zinc ion is four-coordinate in solution [7], while the ethylene glycol solvated zinc ion has neither been studied in solution nor in the solid state. This strongly indicates that complex formation between hexasolvated zinc ions and iodide causes a coordination change, from octahedral to tetrahedral structure, and at the step of the coordination change, in this case at the formation of the first complex, this particular complex will be suppressed (Figs. S1 and S2). On the other hand, when the solvated zinc ion already has a tetrahedral structure a substitution reaction takes place and the first complex will get a well-defined dominance region (Fig. S2). The stability of the solvated diiodozinc complex in solvents where the zinc ion has octahedral structure is also shown by the presence of reported solid state structures, [ZnI_2_(solv)_2_] for solv = water, DMSO, DMF, DMA, acetonitrile and pyridine (Table S3) among the solvents where complex formation data are available for the zinc iodide system; the structures of triiodo- and tetraiodozincate(II) ions in the solid state are summarized in Table S4. Furthermore, no ZnI(solv)_*n*_]^+^ complexes have been reported in the solid state with solvents forming octahedral zinc solvates. However, three solid state structures with a complex with the composition [ZnI(solv)_3_]^+^ have been reported [14–16]. For only one of these solvents, 1-methyl-2(3*H*)-imidazolinethione, the zinc solvate has been characterized to be four-coordinated in a tetrahedral fashion [17]. For the other two solvents, 3{5}-*tert*-butylpyrazole and piperidine, one may assume that they are sufficiently space demanding to have a coordination number lower than six or form sufficiently covalent interactions, also promoting low coordination numbers, to form solvated zinc ions with a lower coordination number than six (Fig. 1).

**Fig. 2:**
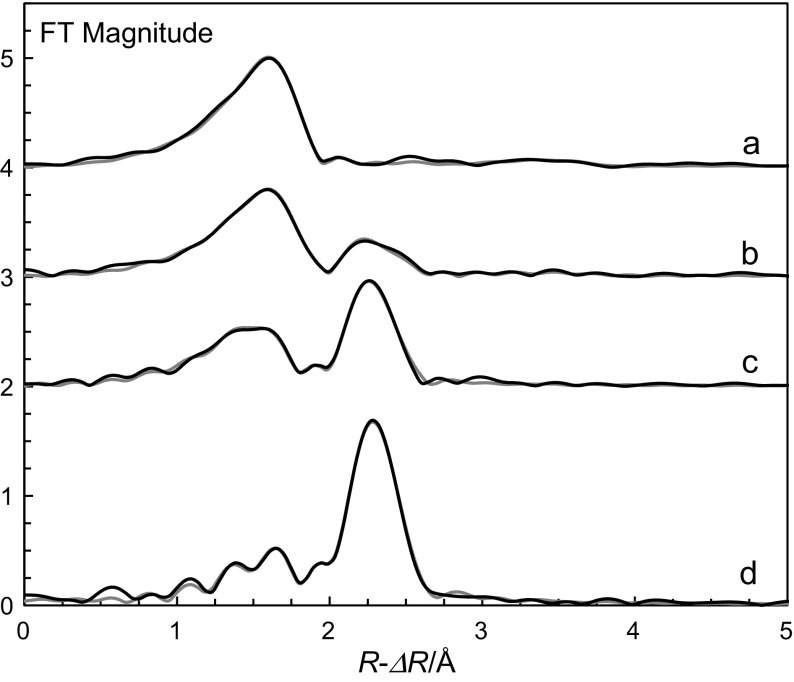
Fit of the Fourier transform of for, *a* solution Zn_0, *b* solution Zn_1, *c* solution Zn_2 and *d* solution Zn_4; experimental data—black, line and model with parameters from Table 3—grey line; the solution compositions are given in Table 2

The aim of the present study is to determine the structures of the methanol solvated zinc ion and iodozinc complexes in solution using EXAFS spectroscopy. These results will validate the assumptions made from the complex formation studies, whether the structure changes from the octahedral structure of methanol solvated zinc ion to tetrahedral iodozinc complexes takes place at the formation of the first complex or not (Fig. 2).

## Experimental

### Preparation of Salts and Solutions

Zinc trifluoromethanesulfonate was prepared by slurrying zinc oxide (Merck), ZnO, in distilled water, and trifluoromethanesulfonic acid (Fluka), CF_3_SO_3_H, was added dropwise until the zinc oxide was dissolved. The solution was filtered and the excess of acid and water were boiled off in an oven at ca. 450 K, and Zn(CF_3_SO_3_)_2_ was obtained as a white powder. It was carefully grained and stored in oven at ca. 450 K.

The methanol solutions were prepared by dissolving weighed amounts of anhydrous zinc trifluoromethanesulfonate, Zn(CF_3_SO_3_)_2_, and zinc iodide (Merck), ZnI_2_, in freshly distilled methanol. The composition of the studied solutions, and their abbreviations are summarized in Table 2.

**Table 2:** Composition of the methanol solutions studied by EXAFS at 25 °C in mol·dm^−3^

Solution	[Zn^2+^]	[I^−^]	[CF_3_SO_3_^−^]	[Na^+^]
Zn_0	0.500		1.000	
Zn_1	0.500	0.500	0.500	
Zn_2	0.500	1.000		
Zn_4	0.500	2.000		1.000

### EXAFS Data Collection and Treatment

X-ray absorption data were collected at the Stanford Synchrotron Radiation Lightsource (SSRL), using the unfocussed 8-pole wiggler beam line 4–1 (old station). SSRL was operated at 3.0 GeV and a ring current of 30–100 mA. The radiation was monochromatized by a Si[220] double crystal monochromator. The monochromator was detuned to 50% of maximum intensity to reduce higher order harmonics. The data were collected in the transmission mode using ion chambers with a gentle flow of nitrogen. For each sample 5 scans were collected and averaged.

The EXAFS oscillations were extracted from averaged raw data using standard procedures for pre-edge subtraction, spline removal and data normalization. In order to obtain quantitative information of the coordination structure of the zinc complexes, the experimental *k*^3^-weighted EXAFS oscillations were analyzed by linear least-squares fits of the data to the EXAFS equation, refining the model parameters: number of backscattering atoms, *N*, mean interatomic distances *R*, Debye–Waller factor coefficients, *σ*^2^, and relative ionization energy, Δ*E*_o_. Data treatment was performed using the EXAFSPAK program package [18]. The energy scale of the X-ray absorption spectra were calibrated by assigning the first inflection point of a metallic zinc foil as 9659 eV [19]. The sample cells were made of 1.5 mm Teflon spacers and with 6 μm polyprolyene X-ray film windows held together with titanium frames. The refinements of the structure parameters were performed with the EXAFSPAK [18] and FEFF7 [20] software packages allowing the determination of the parameters of the local structure around zinc.

The standard deviations reported for the obtained refined parameters listed in Table 3 are those related to the least-squares refinements and do not include any systematic errors. Variations in the refined parameters obtained using different models and data ranges indicate that the accuracy of the distances given for the separate complexes is within ± 0.005–0.02 Å, which is typical for well-defined interactions.

**Table 3:** Mean bond distances, *d*/Å, number of distances, *N*, and Debye–Waller coefficients, *σ*^2^/Å^2^, threshold energy, *E*_o_, amplitude reduction factor, $S_{\text{o}}^{2}$, and the goodness of fit as defined in Ref. [18] from EXAFS studies of the methanol solvated zinc(II) ion and zinc(II) iodide complexes in methanol solution at room temperature

Species						
Interaction	*N*	*d*	*σ* ^*2*^	*E*_o_	$S_{\text{o}}^{2}$	*F*
*Solution Zn_0, 0.50* *mol*·*dm*^−*3*^ *Zn(CF*_*3*_*SO*_*3*_*)*_*2*_ *in methanol*						
[Zn(CH_3_OH)_6_]^2+^						
Zn–O	6	2.071(1)	0.0082(1)	10.3(2)	0.77(1)	8.9
MS (ZnO_6_)	3 × 6	4.15(1)	0.019(2)			
*Solution Zn_1, 0.25* *mol*·*dm*^−*3*^ *Zn(CF*_*3*_*SO*_*3*_*)*_*2*_ *and 0.25* *mol*·*dm*^−*3*^ *ZnI*_*2*_ *in methanol*						
44% [Zn(CH_3_OH)_6_]^2+^] + 12% [ZnI(CH_3_OH)_3_]^+^ + 44% [ZnI_2_(CH_3_OH)_2_]						
Zn–I	1.0(1)	2.538(1)	0.0058(1)	10.4(2)	0.76(1)	9.0
Zn–O	3.9	2.049(1)	0.0094(2)			
*Solution Zn_2, 0.50* *mol*·*dm*^−*3*^ *ZnI*_*2*_ *in methanol*						
[ZnI_2_(CH_3_OH)_2_]^2+^						
Zn–I	2.0(1)	2.545(1)	0.0055(1)	10.6(2)	0.96(1)	10.8
Zn–O	2.0	1.984(2)	0.0094(3)			
*Solution Zn_4, 0.50* *mol*·*dm*^−*3*^ *ZnI*_*2*_ *and 1.00* *mol*·*dm*^−*3*^ *NaI in methanol*						
[ZnI_2_(CH_3_OH)_2_]^2+^						
Zn–I	2.0(2)	2.556(1)	0.0047(1)	10.6(2)	0.86(1)	11.1
Zn–O	2.0	1.991(3)	0.0090(8)			

## Results and Discussion

The Zn–O bond distance in the methanol solvated zinc ion in solution, solution Zn_0, has been determined to be 2.071(2) Å, which is in reasonable agreement with the distance in solid hexakis(methanol)zinc hexafluorosilcate, [Zn(OHCH_3_)_6_]SiF_6_, 2.086 Å [21]. A similar distance has been reported in the hexakis(ethanol)zinc ion in the solid state, 2.079 Å [21]. The obtained Zn–O bond distance in the methanol solvated zinc(II) ion shows that it is six-coordinated, and the multiple scattering pattern confirms an octahedral configuration.

The methanol solvated diiodozinc complex is the dominant species in solutions Zn_2 and Zn_4 (Fig. S1). The Zn–I and Zn–O bond distances have been refined to 2.545(2) and 1.984(4) Å, and 2.556(2) and 1.991(3) Å in solutions Zn_2 and Zn_4, respectively. This strongly indicates that the methanol solvated diiodozinc(II) complex is basically tetrahedral as also found for the hydrated, DMSO, DMA, acetonitrile and pyridine solvated diiodozinc complexes in the solid state (Table S3).

Solution Zn_1 contains ca. 44% of Zn^2+^ and ZnI_2_ each and 12% ZnI^+^ complexes. The observed Zn–I bond distance, 2.538 Å, is in agreement the ones found in solutions Zn_2 and Zn_4, which are dominated by the tetrahedral [ZnI_2_(CH_3_OH)_2_] complex. If the methanol solvated ZnI^+^ complex had remained octahedral a longer Zn–I bond distance should be expected in the order of 2.8 Å, based on the ionic radii given by Shannon [22]. Even though the fraction of the ZnI^+^ complex in solution is expected to be low, ca. 12%, the expected mean Zn–I bond distance should be in the order of 2.58 Å. The observed mean Zn–I bond distance, 2.538 Å, in solution Zn_1, indicates, on the other hand, that the Zn–I bond distance in the [ZnI(CH_3_OH)_3_]^+^ complex is slightly shorter than in [ZnI_2_(CH_3_OH)_2_].

## Conclusions

The methanol solvated zinc ion is six-coordinate in octahedral fashion with a mean Zn–O bond distance of 2.071(2) Å. The methanol solvated ZnI_2_ complex has tetrahedral configuration with mean Zn-I and Zn–O bond distances of 2.55(1) and 1.99(1) Å, respectively. The mean Zn-I bond distance in a solution containing a maximal content of ZnI^+^, ca. 12%, strongly indicates that the first complex also has tetrahedral structure as indicated by the suppressed dominance of the first complex.

## Electronic supplementary material

Below is the link to the electronic supplementary material.

Supplementary material 1 (DOCX 175 kb)
