# Supplementary material for: Structure of Methanol Solvated Iodozinc(II) Complexes in Solution
Source: J Solution Chem. 2018 Mar 19;47(3):560–7. doi: 10.1007/s10953-018-0737-9 (PMC5871641; doi:10.1007/s10953-018-0737-9)
Supplement: Supplementary file 1 — Supplementary material 1 (DOCX 175 kb) [file 10953_2018_737_MOESM1_ESM.docx]

**Structure of Methanol Solvated Iodozinc Complexes in Solution**

Ingmar Persson

Department of Molecular Sciences, Swedish University of Agricultural Sciences, P.O.Box 7015, SE-750 07 Uppsala, Sweden.

**Supporting Material**

***Figure S1***. Complex distribution function for the zinc(II)-iodide system in methanol; black line – Zn^2+^, green line – ZnI^+^, and blue line – ZnI_2_, dashed orange line – composition of solution Zn_1, dashed brown line – composition of solution Zn_2, and dashed purple line – composition of solution Zn_4. The solution composition is given in Table 2, and the graph is based on the stability constants reported in ref. 2.

***Figure S2***. Complex distribution function for the zinc(II)-iodide system in water, dimethylsulfoxide (DMSO), *N,N*-dimethylformamide (DMF), *N,N*-dimethylacetamide (DMA), acetonitrile, hexamethylphosphoric triamide (HMPA) and ethylene glycol; black line – Zn^2+^, green line – ZnI^+^, blue line – ZnI_2_, blue line – ZnI_3_^-^, and orange line – ZnI_4_^2-^. The graphs are based on the stability constants reported in refs. 1 (water), 5 (DMSO), 3 (DMF), 4 (DMA), 7 (HMPA) and 8 (ethylene glycol) in main paper.

***Figure S3***. Complex formation function for the zinc(II)-iodide system in methanol. The graph is based on the stability constants reported in ref. 2 in main paper.

***Table S1***. Transfer thermodynamics of the zinc(II) and iodide ions from water to methanol. Acetonitrile, dimethylsulfoxide and pyridine.

*ΔG*_tr_^o^ (I^-^) ^a^ *ΔH*_tr_^o^ (I^-^) ^b^ *ΔG*_tr_^o^ (Zn^2+^) ^c^ *ΔH*_tr_^o^ (Zn^2+^) ^c^

Methanol +7.4 -1.7 +48.8 -46.8

Dimethylsulfoxide +9.1 -11.8 -48.9 -63.4

Acetonitrile +19.4 -6.8 +55.3 -34.4

Pyridine +19.4 -8.0 -2.3 -85.6

^a^ Johnsson, M.; Persson, I. *Inorg. Chim. Acta* **1987**, *127*, 15-24.

^b^ Johnsson, M.; Persson, I. *Inorg. Chim. Acta* **1987**, *127*, 25-34.

^c^ Chaudhry, M.; Dash, K. C.; Kamienska-Piotrowicz, E.; Kinjo, Y.; Persson, I. *J. Chem. Soc., Faraday Trans.* **1994**, *90*, 2235-2243.

# *Table S2.* Summary of solid state structures of methanol, dimethylsulfoxide, *N,N*-dimethylformamide, *N,N*-dimethylacetamide and acetonitrile solvated zinc ions.

***Methanol***

CIRJIW 2.086 Å Sudbrake, C.; Muller, B.; Vahrenkamp, H., *Eur. J. Inorg. Chem.* **1999**, 2009-2012. [Zn(CH_3_OH)_6_]SiF_6_.

## Dimethylsulfoxide

BIPMIW 2.110 Å Persson, I. *Acta Chem. Scand., Ser. A* **1982**, *36*, 7-13. [Zn(OS(CH_3_)_2_)_6_](ClO_4_)_2_.

BIPMIW01 2.103 Å Chan, E. J.; Cox, B. G.; Harrowfield, J. M.: Ogden, M. I.; Skelton, B W.; White, A. H. *Inorg. Chim. Acta* **2004**, *357*, 2365-2373. [Zn(OS(CH_3_)_2_)_6_](ClO_4_)_2_.

MINGIB 2.111 Å [Garzón-Tovar](http://scripts.iucr.org/cgi-bin/citedin?search_on=name&author_name=Garzon%2DTovar%2C%20L%2E), L.; [Duarte-Ruiz](http://scripts.iucr.org/cgi-bin/citedin?search_on=name&author_name=Duarte%2DRuiz%2C%20A%2E), [Á.;](http://scripts.iucr.org/cgi-bin/citedin?search_on=name&author_name=Duarte%2DRuiz%2C%20A%2E) [Fanwick](http://scripts.iucr.org/cgi-bin/citedin?search_on=name&author_name=Fanwick%2C%20P%2EE%2E), P. E. *Acta Crystallogr., Sect. E* **2013**, *69*, m618-m618. [Zn(OS(CH_3_)_2_)_6_]I_4_.

QUTYOU 2.075 Å Yue, Q.; Sun, Q.; Cheng; A.-L.; Gao, E.-Q. *Cryst. Growth. Des.* **2010**, *10*, 44-47. [Zn(OS(CH_3_)_2_)_6_]Zn_48_O_12_(OH)_2_·111(CH_3_)_2_SO·103H_2_O.

SAWTEQ 2.112 Å Clegg, W.; Elsegood, M. R. J. **2005**, CCDC code 283652. [Zn(OS(CH_3_)_2_)_6_](CF_3_SO_3_)_2_.

***N,N-Dimethylformamide***

HUXGUC 2.104 Å Wang; J.-P.; Wu, Q.; Niu, J.-Y. *Wuji Huaxue Xuebao* **2002**, *18*, 957-. [Zn(OCHN(CH_3_)_2_)_6_](H_2_W_12_SiO_40_)_2_.H_2_O.OCHN(CH_3_)_2_.

***N,N-Dimethylacetamide***

TEDGUG 2.169 Å Yang, G.-S.; Li, M.-N.; Li, S.-L.; Lan, Y.-Q.; He, W.-W.; Wang, X.-L.; Qin, J.-S.; Su, Z.-M. *J. Mater. Chem.* **2012**, *22*, 17947-17953. [Zn(OCCH_3_N(CH_3_)_2_)_6_]·12(CH_3_)_2_NCHO·5CH_3_OH.

# Nitrogen donor solvents

***Acetonitrile***

JEPKEV 2.133 Å Akkus, O. N.; Decken, A.; Knapp, C.; Passmore, J. *J. Chem. Cryst.* **2006**, *36*, 321-329. [Zn(NCCH_3_)_6_](AsF_6_)_2_.

YAMCEW Li, Y.; Yeong, H. Y.; Herdtweck, E.; Voit, B.; Kühn, F. E. *Eur. J. Inorg. Chem.* **2010**, 4587-4590. [Zn(NCCH_3_)_6_][Al(OC(CF_3_)_2_C_6_H_5_)_4_]_2_.

# *Table S3.* Summary of solid state structures of hydrated and dimethylsulfoxide, *N,N*-dimethylformamide, acetonitrile and pyridine solvated diiodozinc complexes, [ZnI_2_(solv)_2_].

***Water***

GALKEJ 2.543 + 2.002 Å Hazell, A. *Acta Crystallogr., Sect. C* **1998**, *44*, 445-447. [ZnI_2_(H_2_O)_2_]. I-Zn-I=122.20 ^o^

## Dimethylsulfoxide

FIQBAI 2.554 + 1.996 Å Wilkins, C. J.; Turnball, M. M.; Wakaira, J. *Z. Kristallogr.* **2000**, *215*, 702-706. [ZnI_2_(OS(CH_3_)_2_)_2_]. I-Zn-I=121.18 ^o^

***N,N-Dimethylformamide***

FEXWIO 2.530 + 1.998 Å Hazell, A. *Acta Crystallogr., Sect. C* **1998**, *44*, 445-44x. [ZnI_2_(OCHN(CH_3_)_2_)_2_]. I-Zn-I=122.00 ^o^

FEXWIO01 2.530 + 1.998 Å [Edwards](http://scripts.iucr.org/cgi-bin/citedin?search_on=name&author_name=Edwards%2C%20R%2EA%2E), R. [A.; Easteal](http://scripts.iucr.org/cgi-bin/citedin?search_on=name&author_name=Easteal%2C%20A%2EJ%2E), [A. J.; Gladkikh](http://scripts.iucr.org/cgi-bin/citedin?search_on=name&author_name=Gladkikh%2C%20O%2EP%2E), [O. P.; Robinson](http://scripts.iucr.org/cgi-bin/citedin?search_on=name&author_name=Robinson%2C%20W%2ET%2E), [W. T.; Turnbull](http://scripts.iucr.org/cgi-bin/citedin?search_on=name&author_name=Turnbull%2C%20M%2EM%2E), [M. M.; Wilkins](http://scripts.iucr.org/cgi-bin/citedin?search_on=name&author_name=Wilkins%2C%20C%2EJ%2E), C. J. *Acta Crystallogr., Sect. B* **1998**, *54*, 663-670. [ZnI_2_(OCHN(CH_3_)_2_)_2_]. I-Zn-I=122.00 ^o^

# Nitrogen donor solvents

***Acetonitrile***

TIRLEL 2.532 + 2.038 Å Raubacher, F.; Weller, F. *Z. Kristallogr.* **1996**, *311*, 576-576. [ZnI_2_(NCCH_3_)_2_] I-Zn-I=120.47 ^o^

TIRLEL01 2.532 + 2.046 Å Bhosekar, G.; Jess, I.; Nather, C. *Acta Crystallogr., Sect. E* **2006**, *62*, m1315-m1316. [ZnI_2_(NCCH_3_)_2_] I-Zn-I=120.5 ^o^

***Pyridine***

ZNPYDI 2.552 + 2.055 Å Le Querler, J. F.; Borel, M. M.; Leclaire, A. *Acta Crystallogr., Sect. B* **1977**, *33*, 2299-2300. [ZnI_2_(NC_5_H_5_)_2_] I-Zn-I=120.33 ^o^

# *Table S4.* Summary of solid state structures containing triiodozincate and tetraiodozincate complexes.

# Tetraiodozincate complexes

408796 2.595 Å Friese, K.; Madariaga, G.; Breczewski, T. *Z. Kristallogr.* **1998**, *213*, 591-595. Li_2_[ZnI_4_]

410696 2.597 Å Friese, K.; Neubert, B.; Madariaga, G.; Breczewski, T. *Z. Kristallogr.* **1999**, *214*, 659-665. Cs_2_[ZnI_4_]

HIJTAV 2.599 Å Muller, B.; Vahrenkamp, H. *Eur. J. Inorg. Chem.* **1999**, 137. [Zn(NOC_6_H_5_)_2_I]_2_[ZnI_4_]

59353 2.602 Å Friese, K.; Madariaga, G.; Breczewski, T. *Acta Crystallogr., Sect. C* **1998**, *54*, 1737-1739. Cs_3_[ZnI_4_]I

KEFWEX01 2.605 Å Werk, M. L.; Chapuis, G.; Zuniga, F. J. *Acta Crystallogr., Sect. B* **1990**, *46*, 187. ((CH_3_)_4_N)_2_[ZnI_4_]

BZDAZP 2.606 Å Orioli, P. L.; Lip, H. C. *Cryst. Struct. Commun.* **1974**, *3*, 477. (C_11_H_13_N_2_)_2_[ZnI_4_]

KEFWEX 2.607 Å Hasebe, K.; Asahi, T.; Gesi, K. *Acta Crystallogr., Sect. C* **1990**, *46*, 218. ((CH_3_)_4_N)_2_[ZnI_4_]

56423 2.608 Å Purgahn, J. Ph.D. Thesis, Universität Karlsruhe **1998**, 1-173. Rb_2_[ZnI_4_]

37099 2.613 Å Ammlung, R. L.;Scaringe, R. P.; Ibers, J. A.; Shriver, D. F.; Whitmore, D. H. *J. Solid State Chem.* **1979**, *29*, 401-415. Tl_2_[ZnI_4_]

202056 2.614 Å Louer, M.; Louer, D. *J. Solid State Chem.* **1986**, *65*, 272-279. Cs_3_[ZnI_4_]NO_3_

402062 2.615 Å Zhang, Z.; Lutz, H. D. *Z. Kristallogr.* **1995**, *210*, 691-692. Li_2_[ZnI_4_]

FIGMUE 2.615 Å Grapperhaus, C. A.; Mullins, C. S.; Mashuta, M. S. *Inorg. Chim. Acta* **2005**, *358*, 623. [Zn(N_2_S_2_C_14_H_32_)I]_2_[ZnI_4_]∙0.25CH_3_OH

KIYVAP 2.616 Å Harrison, W. T. A.; Howie, J.; Skakle, J.; Wardell, J. L. *Acta Crystallogr., Sect. C* **2000**, *56*, e124. ((C_2_H_5_)_4_N)_2_[ZnI_4_]

MEGPAQ 2.616 Å Lemmerer, A.; Billing, D. G. *Acta Crystallogr., Sect. E* **2006**, *62*, m779. ((*p*-C_6_H_4_(CH_3_)_2_)[ZnI_4_]∙2H_2_O

SEGSUT 2.617 Å Ishihara, H.; Horiuchi, K.; Svoboda, I.; Fuess, H.; Gesing, T. M.; Buhl, J.-C.; Terao, H. *Z. Naturforsch., Teil B* **2006**, *61*, 69. (C_4_H_12_N_2_)[ZnI_4_]

OBUGOH 2.618 Å [ZnON(C_12_H_9_)_2_I]_2_[ZnI_4_]∙CH_3_CN

KEFWEX02 2.621 Å Hasebe, K.; Asahi, T.; Gesi, K. *Acta Crystallogr., Sect. B* **1990**, *46*, 187. ((CH_3_)_4_N)_2_[ZnI_4_]

FEYLUR 2.624 Å Glavcheva, Z.; Nakanishi, H.; Okada, S.; Umezawa, H. *Mater. Lett.* **2004**, *58*, 2466. (C_8_H_13_N_2_)_2_[ZnI_4_]

FEYLOL 2.625 Å Glavcheva, Z.; Nakanishi, H.; Okada, S.; Umezawa, H. *Mater. Lett.* **2004**, *58*, 2466. (C_7_H_7_N_2_)_2_[ZnI_4_]

KIYVAP01 2.632 Å Harrison, W. T. A.; Howie, J.; Skakle, J.; Wardell, J. L. *Acta Crystallogr., Sect. C* **2000**, *56*, e124. ((C_2_H_5_)_4_N)_2_[ZnI_4_]

69034 2.635 Å Pfitzner, A.; Lutz, H. D.; Cockcroft, J. K. *J. Solid State Chem.* **1990**, *87*, 463-466. Li_2_[ZnI_4_]

**Mean Zn-I 2.613 Å/21 structures**

# Triiodozincate complexes

JOYXEA 2.595 + 2.019 Å Songchun Jin; Nieuwenhuyzen, M.; Wilkins, C. J. *J. Chem. Soc., Dalton Trans.* **1992**, 2071. [ZnI_3_(ONC_5_H_5_)]

XAHVUY 2.571 + 2.068 Å Huebner, L.; Kornienko, A.; Emge, T. J.; Brennan, J. G. *Inorg. Chem.* **2004**, *43*, 5659. [Nd(OC_4_H_8_)_5_I_2_][ZnI_3_(OC_4_H_8_)]

22121 2.571 + 2.069 Å Holinski, A.; Brehler, B. *Acta Crystallogr., Sect. B* **1970**, *26*, 1915-1919. K[ZnI_3_H_2_O]∙H_2_O

**Mean Zn-I 2.579 + 2.052 Å/3 structures**

# Diiodozinc complexes

FIPTAZ 2.512 + 2.015 Å Edwards, R. A.; Gladkikh, O. P.; Nieuwenhuyzen, M.; Wilkins, C. J. *Z. Kristallogr.* **1999**, *214*, 111. [ZnI_2_(OC(CH_3_)(C_6_H_5_))_2_)_2_]

HIKCEJ 2.524 + 2.041 Å Muller, B.; Vahrenkamp, H. *Eur. J. Inorg. Chem.* **1999**, 129. [ZnI_2_(OCH-*m*-C_6_H_4_OCH_3_)_2_]

HIKCAF 2.525 + 2.024 Å Muller, B.; Vahrenkamp, H. *Eur. J. Inorg. Chem.* **1999**, 129. [ZnI_2_(OCH-*p*-C_6_H_5_OCH_3_)_2_]

HIKCUZ 2.529 + 2.046 Å Muller, B.; Vahrenkamp, H. *Eur. J. Inorg. Chem.* **1999**, 129. [ZnI_2_(OCH-*p*-C_6_H_5_)_2_]

TETTUH 2.529 + 1.998 Å Dong Mei-Bin, Li Song-Xian, Zhang Han-Hui, Yong Jing-Hai, Geng Yi-Zhi, Xu Ji-Yan *Jiegou Huaxue* **1996**, *15*, 311. [ZnI_2_(ONC_5_H_3_(CH_3_)(NO_2_))_2_]

FEXWIO 2.530 + 1.998 Å Roshchupkina, O. S.; Bulatov, A. V.; Samovarov, Y. K.; Slovokhotov, Y. L.; Struchkov, Y. T. *Koord. Khim.* **1987**, *13*, 321. [ZnI_2_(OCH(N(CH_3_)_2_)_2_]

HIKCIN 2.532 + 2.046 Å Muller, B.; Vahrenkamp, H. *Eur. J. Inorg. Chem.* **1999**, 129. [ZnI_2_(OCH-*p*-C_6_H_4_CH_3_)_2_]

HIKCIN01 2.532 + 2.046 Å Henrich, M.; Delgado, A.; Molins, E.; Roig, A.; Llebaria, A. *Eur. J. Inorg. Chem.* **1999**, 129. [ZnI_2_(OCH-*p*-C_6_H_4_CH_3_)_2_]

FEXWIO01 2.534 + 1.998 Å Edwards, R. A.; Easteal, A. J.; Gladkikh, O. P.; Robinson, W. T.; Turnbull, M. M.; Wilkins, C. J. *Acta Crystallogr., Sect. B* **1998**, *54*, 663. [ZnI_2_(OCH(N(CH_3_)_2_)_2_]

VIDBOA 2.539 + 1.977 Å Savinkina, E. V.; Buravlev, E. A.; Zamilatskov, I. A.; Albov, D. V. *Acta Crystallogr., Sect. E* **2007**, *63*, m1094. [ZnI_2_(OC(CH_3_)NH_2_))_2_]

SANRUV 2.540 + 1.991 Å Jing-Min Shi, Zhe Liu, Jian-Jun Lu, Lian-Dong Liu *Acta Crystallogr., Sect. E* **2005**, *61*, m856. [ZnI_2_(O-*p*-NC_5_H_4_-CH_3_)_2_]

GALKEJ 2.543 + 2.002 Å Hazell, A. *Acta Crystallogr., Sect. C* **1988**, *44*, 445. [ZnI_2_(OH_2_)_2_)_2_]

FEXWIO02 2.548 + 2.010 Å Edwards, R. A.; Easteal, A. J.; Gladkikh, O. P.; Robinson, W. T.; Turnbull, M. M.; Wilkins, C. J. *Acta Crystallogr., Sect. B* **1998**, *54*, 663. [ZnI_2_(OCH(N(CH_3_)_2_)_2_]

SECHIS 2.550 + 1.972 Å Yong Nie; Pritzkow, H.; Wadepohl, H.; Siebert, W. *J. Organometal. Chem.* **2005**, *690*, 4531. [ZnI_2_(OP(C_6_H_5_)_3_) _2_]

IPNOZN01 2.552 + 1.997 Å Edwards, R. A.; Gladkikh, O. P.; Nieuwenhuyzen, M.; Wilkins, C. J. *Z. Kristallogr.* **1999**, *214*, 111. [ZnI_2_(ONC_5_H_5_)_2_]

FIQBAI 2.554 + 1.996 Å Edwards, R. A.; Gladkikh, O. P.; Nieuwenhuyzen, M.; Wilkins, C. J. *Z. Kristallogr.* **1999**, *214*, 111. [ZnI_2_(OS(CH_3_)_2_)_2_]

ACAQAW 2.558 + 1.975 Å Furmanova, N. G.; Resnyansky, V. F.; Sulaimankulov, K. S.; Zhorobekova, S. Z.; Sulaimankulova, D. K. *Kristallografiya* **2001**, *46*, 58. [ZnI_2_(OC(NH_2_)_2_]

AXUGAB 2.564 + 1.968 Å Popovic, Z.; Roje, V.; Pavlovic, G.; Matkovic-Calogovic, D.; Rajic, M.; Leban, I. Polyhedron 2004, 23, 1293. [ZnI_2_(ONC_18_C_15_)_2_]

IPNOZN 2.567 + 1.983 Å Sawitzki, G.; von Schnering, H. G. *Chem. Ber.* **1974**, *107*, 3266. [ZnI_2_(ONC_5_H_5_)_2_]

VATROX 2.576 + 1.955 Å Bottino, F. A.; Finocchiaro, P.; Libertini, E.; Mattern, G. *Z. Kristiallogr.* **1989**, *187*, 71. [ZnI_2_(OC_10_H_13_N)_2_]

TEGNUO 2.585 + 2.008 Å Zelenak, V.; Gyoryova, K.; Cisarova, I.; Loub, J. *Acta Crystallogr., Sect. C* **1996**, *52*, 1917. [ZnI_2_(OC_8_H_8_N_2_O_2_)_2_]

FIQBIQ 2.606 + 1.940 Å Edwards, R. A.; Gladkikh, O. P.; Nieuwenhuyzen, M.; Wilkins, C. J. *Z. Kristallogr.* **1999**, *214*, 111. [ZnI_2_(ON(CH_3_)_3_)_2_]

**Mean Zn-I 2.547 + 1.999 Å/22 structures**
